# A Simulation Model for Geriatric Education: Theory, Virtual Transformation and Quality Improvement

**DOI:** 10.1093/geroni/igab046.000

**Published:** 2021-12-17

**Authors:** Jennifer Drost, Susan Fosnight

## Abstract

Delivery of effective healthcare for the geriatric population is often complex due to the interplay between physical, social, and emotional variables. It is well established that it is the interplay between chronic medical conditions, social determinants of health, function and geriatric syndromes that drives outcomes. This complexity makes it especially important for the healthcare team to take an interprofessional team approach to avoid fragmented care which can lead to patient dissatisfaction, an ineffective plan of care, and low-quality outcomes. However, effective teamwork is not innate to healthcare; it must be learned and developed over time through purposeful education. The literature on team training supports active learning pedagogies such as simulation-based education that has emerged as an effective way to translate teamwork education into practice. Participation in active learning such as simulation, provides learners with authentic experiences that become cognitive frames that can transition into real practice. Education of adult learners should be a scaffolding of experiences that build on one another. This approach can lead the learner from the acquisition of basic knowledge, skills, and attitudes, to higher levels of competency and clinical judgement. Simulation simultaneously engages cognitive, perceptual-motor, and affective learning, and when combined with effective debriefing can lead to higher levels of learning. Effective models with scaffolding of experiences using simulations for geriatric team training are lacking in the literature. We describe here the theoretical framework for such training, adaptions of in-person and virtual training models due to COVID-19 restrictions through rapid cycle quality improvement.

